# Complex Non-sinus-associated Pachymeningeal Lymphatic Structures: Interrelationship With Blood Microvasculature

**DOI:** 10.3389/fphys.2019.01364

**Published:** 2019-10-31

**Authors:** Olga V. Glinskii, Virginia H. Huxley, Leike Xie, Filiz Bunyak, Kannappan Palaniappan, Vladislav V. Glinsky

**Affiliations:** ^1^Department of Medical Pharmacology and Physiology, University of Missouri, Columbia, MO, United States; ^2^Dalton Cardiovascular Research Center, University of Missouri, Columbia, MO, United States; ^3^Reasearch Service, Harry S. Truman Memorial Veterans Hospital, Columbia, MO, United States; ^4^Center for Gender Physiology and Environmental Adaptation, University of Missouri, Columbia, MO, United States; ^5^Computational Imaging and VisAnalysis Lab, Department of Electrical Engineering and Computer Science, University of Missouri, Columbia, MO, United States; ^6^Department of Pathology and Anatomical Sciences, University of Missouri, Columbia, MO, United States

**Keywords:** dura mater, lymphatics, vascular structures, microvessels, pachymeningeal

## Abstract

The contribution of cranial dura mater vascular networks, as means for maintaining brain fluid movement and balance, and as the source of significant initiators and/or contributors to neurological disorders, has been overlooked. These networks consist of both blood and lymphatic vessels. The latter were discovered recently and described as sinus-associated structures thus changing the old paradigm that central nervous system lacks lymphatics. In this study, using markers specific to blood and lymphatic endothelia, we demonstrate the existence of the complex non-sinus-associated pachymeningeal lymphatic vasculature. We further show the interrelationship and possible connections between lymphatic vessels and the dural blood circulatory system. Our novel findings reveal the presence of lymphatic-like structures that exist on their own and/or in close proximity to microvessels. Of particular interest are sub-sets of vascular complexes with dual (lymphatic and blood) vessel identity representing a unique microenvironment within the cranial dura. The close association of the systemic blood circulation and meningeal lymphatics achieved in these complexes could facilitate fluid exchange between the two compartments and constitute an alternative route for CSF drainage.

## Introduction

The dura mater is the most external meningeal layer surrounding the central nervous system. This tissue was traditionally viewed as a protective fibrous covering that contains venous sinuses but lacks any additional functions. However, detailed anatomical and embryological studies revealed that the cranial dura is in fact, highly vascularized (Coles et al., 2017a). Indeed, our recent data demonstrate that large areas of the dura mater connective tissue stroma possess extensive networks of blood microvasculature (Glinskii et al., 2007, 2008, 2013; Bunyak et al., 2008; Pelapur et al., 2014; Prasath et al., 2015; Kassim et al., 2016a, b; Meena et al., 2016). This tissue is also densely innervated (Coles et al., 2017a) and possesses an exceptionally structured collagenic architecture (Protasoni et al., 2011). The venous vessel organization is particularly complex in cranial dura (Roland et al., 1987) with two distinct venous drainage systems. One, “well-ordered,” is comprised of two satellite veins that accompany the principal superficial artery located in the periosteal layer of the dura mater (Roland et al., 1987). The other, “apparently anarchic” (Roland et al., 1987) is situated within the meningeal layer of cranial dura and characterized as an irregular arrangement which may come together as plexuses (including inner vascular plexus). This second system appears to play a role in cerebrospinal fluid (CSF) absorption (Dandy, 1929; Mack et al., 2009). Positioned inside the intracranial cavity, an indistensible closed space, the dura mater not only protects intracranial structures, but also represents the critical site for CSF turnover (Dandy, 1929; Papaiconomou et al., 2004; Mack et al., 2009) and the main route for the brain venous/CSF outflow. Further, given these anatomical traits and location, there is a reason to believe that alterations within vascular networks into, and particularly out of the CNS within the cranial dura mater are significant, yet unappreciated initiators and/or contributors to a myriad neurological disorders including migraine (Glinskii et al., 2017), dural aneurysms (Baltsavias et al., 2015a, b) leading to a higher risk of intracranial hemorrhage, dural sinus/cerebral vein thrombosis (Baltsavias et al., 2015a, c), multiple sclerosis (Louveau et al., 2016), and Alzheimer’s disease (Louveau et al., 2016).

Recent rediscovery of dura mater sinus-associated lymphatic vessels has changed the centuries-old paradigm that the CNS is devoid of a lymphatic system and added even more complexity to the structural and functional pachymeningeal vascular makeup (Aspelund et al., 2015; Louveau et al., 2015). Even though the first description of human meningeal lymphatic vessels could be traced back to the end of the XVIII century, followed by sporadic reports of meningeal lymphatic structures in other mammalian species, until recently this literature was largely overlooked or ignored [reviewed in references Da Mesquita et al. (2018) and Sandrone et al. (2019)]. Newly identified lymphatic vessels within the dura mater (Louveau et al., 2015) suggest that constant immune surveillance of the CNS occurs within the cranial space (Kipnis et al., 2012; Ransohoff and Engelhardt, 2012; Shechter et al., 2013). It was shown that sinus-associated meningeal lymphatic vessels drain out of the skull alongside the blood vessels and cranial nerves, functioning as a direct clearance route for brain interstitial fluid, macromolecules, and CSF into the deep cervical lymph nodes (Louveau et al., 2015). Extensive contemporary studies, focusing with CSF drainage and the meningeal compartment suggest that “cervical lymph nodes provide an intermediate way-station for fluid traveling from the brain interstitial space to the systemic circulation” (Iliff et al., 2015). A critical need remains, though, for a comprehensive understanding of the anatomical and functional relationship between these dura lymphatic vessels and the systemic blood microvasculature. In this study we demonstrate the existence of the variety of previously unknown meningeal lymphatic structures. Features distinctive to these vessels are that they are not sinus-associated and include unique and specific anatomic structures within the vascular networks with dual, blood, and lymphatic vessel identity revealing a possible alternative pathway for pachymeningeal lymphatic access to the systemic circulation.

## Materials and Methods

### Animals

All animal experimental procedures were approved by the University of Missouri Institutional Animal Care and Use Committee. Mice (2–4 month-old female C57BL/6J, wild type, and Prox1^eGFP^ transgenic, in which lymphatic vessels exhibit bright fluorescence due to eGFP expression under the direction of a Prox1 promotor). In this study, 5 wild type C57BL/6J (3 female, 2 male) animals and 3 Prox1^eGFP^ transgenic (1 female, 2 male) mice were used. The Prox1^eGFP^ transgenic mouse was originally developed by the group of Dr. Young-Kwon Hong and first reported in 2011 (Choi et al., 2011). The model was extensively validated by the authors by cross-staining with other endothelial cell markers in an array of mouse tissues and organs. It was shown to faithfully recapitulate the expression pattern of the Prox1 gene in lymphatic endothelial cells and other Prox1-expressing organs, and enabled convenient visualization of detailed structure and morphology of lymphatic vessels and networks. The model was further validated and successfully used by multiple labs (at least 65 citations in PubMed Central). In addition, as not all lymphatic-like structures express Prox1, we have used lymphatic vessel staining with anti-LYVE-1 antibody in wild type C57BL/6J mice coupled with blood vessel visualization by fluorescently labeled soybean agglutinin (SBA) lectin. Using two independent models (Prox1^eGFP^ and wild type C57BL/6J) is also reducing possibilities for potential imaging artifacts and adds more validity to the obtained results.

### Transcardial Perfusion

Immediately following sacrifice, the chest cavity of the mouse was carefully opened making sure that no significant blood vessels were damaged. A small incision in the right atrial chamber was made to provide an outlet for the blood and/or fluid during perfusion. A 26 gauge needle connected to the syringe filled with prewarmed to 37°C Kreb’s/BSA solution was inserted into the left ventricle chamber and the entire body of the animal was perfused through the heart with 2 ml of Kreb’s/BSA solution at the rate of ∼3 ml/min to wash out the blood. Of note, this perfusion rate is at least fivefold less than the average cardiac output in mice, which is important for ensuring that no damage is done to the vasculature due to the exceeding perfusion pressure. Next, wild type animals were perfused with 1 ml of Kreb’s/BSA containing AlexaFluor 488-conjugated anti-mouse Lyve-1 antibody (eBioscience, clone ALY7 rat IgG1 kappa, MW ∼150 kDa, Cat # 53-0443-82, 1:100 final dilution) and 20 μg/ml AlexaFluor 594-conjugated soybean agglutinin (SBA) lectin (Thermo Fisher Scientific, MW ∼120 kDa Cat # L32462) to stain and identify lymphatic structures and blood dura microvessels, respectively. In case of Prox1^eGFP^ mice, only AlexaFluor 594-conjugated SBA lectin was used for transcardial perfusion at the same concentration.

### Tissue Preparation, Image Acquisition, and Analysis

The cranial skull together with the dura mater was removed, mounted on the microscope slide using the optimum cutting temperature (OCT) compound (VWR, Chicago, IL, United States, Cat # 25608-930) and freshly imaged without fixation. Microvascular networks were imaged using spinning disk confocal automated IX81 inverted microscope with Olympus IX2-DSU unit, Xenon light source, high QE monochrome CCD camera for linear 3D collection and rendering, and Slidebook^TM^ software.

The 15 to 40 μm thick Z-stacks were acquired with 2 μm step size. Image analysis of 3D Z-stacks was performed using ImageJ version 1.44o software. In addition, the image in Figure 5B was modified using a customized pixel level and local image processing algorithms written in Matlab to reduce background stain, improve the signal-to-noise ratio (denoising) and remove the spatially varying blur by fusing together multi-focus epifluorescence dual stain microscopy image slices (Pelapur et al., 2014; Prasath et al., 2015). The non-linear filtering algorithm was used to more clearly reveal the spatial relationships between blood vessels and lymphatics vasculature. These image filtering and processing algorithms were originally developed for automatic segmentation of the single stain blood vessel microvasculature imagery (Kassim et al., 2016b) and were extended to work with the dual stain blood vessel and lymphatics imagery in this paper. Each channel (red and green) was enhanced independently at each focal plane to remove spatially varying blur in each image slice. All of the Z-stack images were combined into a single fused multi-focus image for each color channel separately. The fused layers were then recombined into an enhanced red-green dual channel color image. This enabled us to enhance the vascular structures of blood and lymphatic vessel stains independently. As a result, a single fused, enhanced, deconvolved image was created, in which all structures in different parts of the image became visible and more sharply in-focus.

**FIGURE 1:**
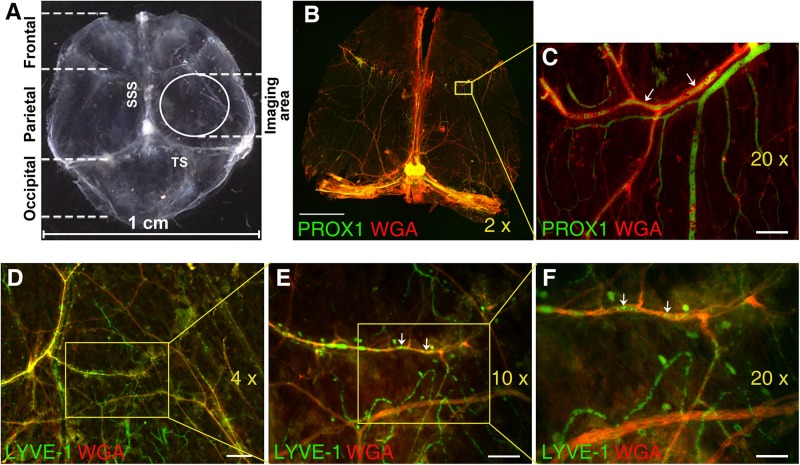
Immunofluorescence analysis of mouse dura mater non-sinus-associated blood and lymphatic microvessels. **(A)** Isolated mouse dura mater completely separated from the skull. SSS, superior sagittal sinus; TS, transverse sinus. **(B)** Stitched image of the whole mount of Prox1eGFP mouse dura matter immunostained with AlexaFluor 594-conjugated WGA lectin (red) to visualize blood vessels and imaged using 2× objective. Yellow inset outlines the area imaged with 20× objective and shown in **(C)**. **(C)** Detailed view of PROX1-positive lymphatic vessels (green) and WGA-positive blood vessels (red) in parietal area of dura mater. **(D)** Through **(F)** Immunofluorescence images of dura mater blood and lymphatic microvasculature in parietal area of wild type mouse dura mater acquired with 4× **(D)**, 10× **(E)**, and 20× **(F)** lenses. Isolated mouse dura mater was stained with anti-LYVE-1 antibody (green) for lymphatic and WGA lectin (red) for blood microvessels. In **(C,E,F)** please note green structures within red stained blood vessels (arrows), which could represent potential artifacts due to auto fluorescence of blood cells trapped in the vasculature. Scale bars, 2000 μm **(B)**, 50 μm **(C)**, 200 μm **(D)**, 100 μm **(E)**, and 50 μm **(F)**.

In select experiments (Figures 1B,C), immediately after animal sacrifice, Prox1^eGFP^ mouse DM was completely separated from the skull. Tissue was fixed in 10% neutral buffered formalin solution (Sigma, Cat # HT501320). After 24 h isolated dura was washed with PBS and permeabilized with 0.5% Triton X-100 for 30 min. Non-specific sites were masked with blocking reagent (3% BSA in PBS) and blood vessels were stained with wheat germ agglutinin (WGA) lectin conjugated with AlexaFluor 594 (Thermo Fisher Scientific, Cat # W11262, 25 μg/ml). In our experience WGA lectin exhibit superior tissue penetration, selective blood vessel staining, and better signal-to-noise ratio in immunohistochemistry (IHC) applications. Flat mounts were prepared and mounted on a slide using ibidi Mounting Medium for fluorescence microscopy (ibidi Inc., Fitchburg, WI, United States Cat # 50001). The whole mounts were imaged with 2× lens and stitched (Figure 1B) using Keyence BZ-X800 microscope (Keyence Corporation of America, Itasca, IL, United States) using GFP and Texas Red channels. In addition, 20× images were acquired using the same channels (Figure 1C). Similarly, in wild type mice, dura mater was isolated, processed as above and probed with anti-LYVE-1 antibody conjugated with AlexaFluor 488 (eBioscience, Cat # 53-0443-82, dilution 1:25) and WGA lectin conjugated with AlexaFluor 594 (Thermo Fisher Scientific, Cat # W11262, 25 μg/ml). Flat mounts were prepared and mounted on a slide using ibidi Mounting Medium for fluorescence microscopy (ibidi United States, Inc., Fitchburg, WI, Cat # 50001). Slides were examined using a fluorescent Olympus IX2-DSU microscope equipped with 4×, 10×, and 20× lenses (Figures 1D–F).

In control experiments (Supplementary Figures S1A–D; non-perfused C57BL/6J wild type mouse auto fluorescence control), DM was isolated, prepped, fixed and permeabilized as above, however, was not stained with any fluorophores. In perfused wild type mouse auto fluorescence control (Supplementary Figures S1E–H), immediately after sacrifice, the mouse was perfused via left ventricle with 2.5 ml of prewarmed to 37°C Kreb’s/BSA solution followed by 1.5 ml of 4% formaldehyde solution in PBS. The sample was not stained with any fluorophores and mounted on the slide as above. In Prox1^eGFP^ mouse auto fluorescence controls (Supplementary Figures S1I–P), the samples were counterstained with WGA lectin conjugated with AlexaFluor 594 to allow for distinguishing between green auto fluorescence of systemic blood vessels and GFP positivity of lymphatic vessels. All controls were imaged using the same filter cubes and settings as experimental samples.

## Results and Discussion

Microscopic imaging of meningeal microvasculature in mice presents significant technical challenges. Because mouse dura mater is extremely thin, invasive dissection of murine meninges with their unsupported membranes can alter tissue architecture. Mouse meninges architecture defined by very delicate membranes is practically impossible to preserve if the overlying skull is removed (Coles et al., 2017b). For these reasons, in most experiments we have chosen to image pachymeningeal microvascular networks while dura mater is still attached to the skull. The parietal area of dura mater was selected to image non-sinus-associated vascular structures in this study (Figure 1A).

Nonetheless, in select experiments (Figure 1) mouse dura was completely isolated from the skull and subjected to IHC analysis using WGA lectin to visualize blood vessel in Prox1^eGFP^ mice (Figures 1B,C), or anti-LYVE-1 antibody and WGA lectin to visualize lymphatic structures and blood vessel, respectively, in wild type mice (Figures 1D–F). Even on the images acquired using 2×, 4×, and 10× lenses extensive blood microvascular and non-sinus-associated lymphatic structures could be appreciated (Figures 1B–F). However, the use of 20× lens (Figure 1D) allows for a more detailed analysis of microvessel morphology and interrelationship. One potential problem here is that some of the structures observed within blood vasculature and exhibiting green fluorescence signal could potentially present artifacts due to auto fluorescence of blood cells remaining in the vessels (Figures 1C,E,F, arrows). Our control auto fluorescence experiments (Supplementary Figure S1) demonstrate that transcardial perfusion efficiently eliminates this potential problem. Thus, the rest of the images shown in this study were acquired on unfixed dura mater preparations obtained from experimental animals following transcardial perfusion.

**FIGURE 2:**
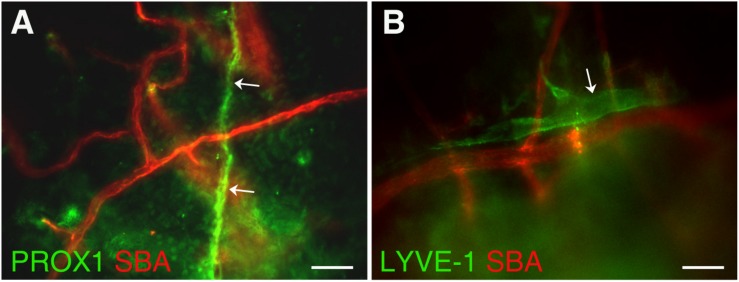
Presence of lymphatic vessels of differing sizes and degrees of association with meningeal blood vasculature. **(A)** Lymphatic microvessel (Prox-1, green, white arrows) ∼5 μm in diameter exhibiting no apparent association with blood vessels (SBA lectin, red). **(B)** Lymphatic vessel (LYVE-1, green, white arrow) ∼25 μm in diameter runing along side an ∼20–25 μm diameter arteriole (SBA lectin, red). In panel **(A)** Prox1^eGFP^ mice, in which lymphatic vessels exhibit bright green fluorescence, the hearts were injected with Kreb’s/albumin solution containing AlexaFluor 594-conjugated SBA lectin to visualize the systemic blood vasculature. In **(B)** wild type C57BL/6J mice were perfused through the heart with Kreb’s/albumin solution containing AlexaFluor 488-conjugated anti-mouse Lyve-1 antibody and AlexaFluor 594-conjugated SBA lectin to visualize lymphatic and blood vasculatures, respectively. In **(A,B)** the images, acquired using spinning disk confocal microscopy, are the sum slices of the Z stack projection. Scale bars, 50 μm.

In this study, we investigated the detailed structure of parietal pachymeningeal vascular networks using markers specific to blood and lymphatic endothelia. Our focus was on the relationship, and possible connections, between the lymphatic and blood microvasculature. Dual labeling not only revealed various non-sinus-associated lymphatic structures within cranial dura, but also demonstrated that they are separate and distinct from blood vessels. We found that the parietal dura contains a complex and diverse lymphatic vasculature. It includes lymphatic vessels of different sizes ranging from small capillaries [diameter ∼5 μm (Figure 2A)] to vascular structures of much larger sizes [up to ∼25 μm (Figure 2B)]. These lymphatic vessels located in parietal area exhibit contrasting relationship with the blood vasculature: running individually, without any association with blood vessels (Figure 2A); or alongside the small arterioles (Figure 2B).

**FIGURE 3:**
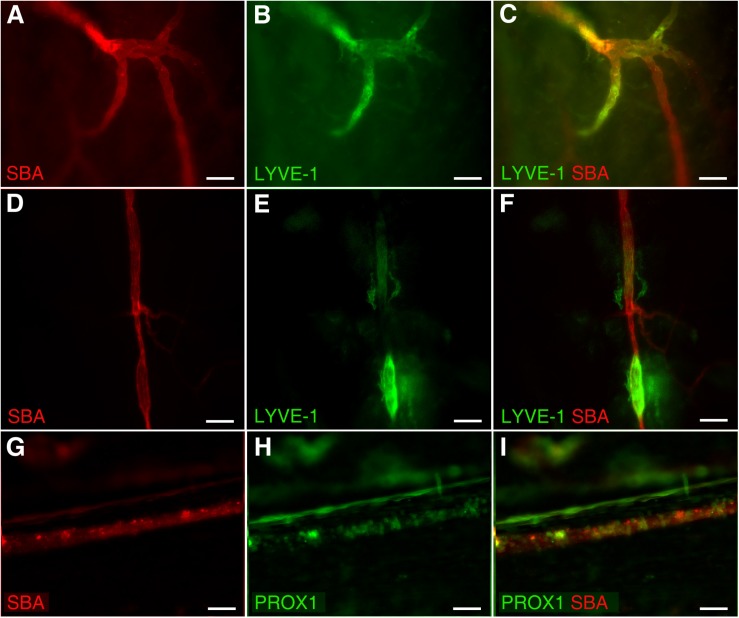
Dual vessel specification within the same vascular network or a single vessel. Blood and lymphatic structures were visualized by spinning disk confocal microscopy in wild type C57BL/6J mice by transcardial perfusion with Kreb’s/albumin solution containing AlexaFluor 594-conjugated SBA lectin and AlexaFluor 488-conjugated anti-mouse Lyve-1 antibody, respectively. **(A–C)** Significant parts of the microvascular network exhibit both blood (SBA lectin, red) and lymphatic (LYVE-1, green) identity. **(D–F)** A single blood (SBA lectin, red) microvessel with the segments exhibiting lymphatic (LYVE-1, green) identity as well. **(G–I)** Mosaic SBA lectin (red)/Prox1 (green) positivity pattern within a single vessel in Prox1^eGFP^ mice. To visualize the blood vasculature, Prox1eGFP mice, in which lymphatic vessels exhibit bright green fluorescence, the hearts were perfused with Kreb’s/albumin solution containing AlexaFluor 594-conjugated SBA lectin. In **(A,D,G)** SBA lectin staining (red). In **(B,E)** anti-LYVE-1 staining (green). In **(H)** Prox1 signal (green). In **(C,F,I)** superimposed images. Scale bars 50 μm.

In addition, our data revealed unique dural microvascular networks exhibiting dual vessel specification suggesting the convergence of branches with blood and lymphatic vessel identity into one common network (Figures 3A–C). This remarkable feature could be observed even in different segments of a single vessel in wild type animals with LYVE-1/SBA staining (Figures 3D–F), or present as “mosaic” SBA lectin/Prox1 positivity pattern within a single vessel in Prox1^eGFP^ mice (Figures 3G–I). This discovery led us to speculate that dural vascular networks could perform dual functions supporting both brain venous outflow and CSF absorption. Given that both SBA staining (blood vessel) and LYVE-1 staining (lymphatic vessel) are present in these structures it would appear that they possess the ability to be sites of plasticity, where the function can switch from one compartment to the other. Whether these streams actually merge or interact requires closer scrutiny. However, in any case the fluids in the two compartments are brought here into close apposition possibly providing a mechanism whereby these vascular networks could contribute to CSF absorption as well. Indeed, CSF reabsorption into the venous system occurs not only by the arachnoid granulations or villi, but also through the dural deep venous plexus (Dandy, 1929; Osaka et al., 1980; Papaiconomou et al., 2004; Mack et al., 2009). This could be particularly important during development of the CSF pathway in human embryos (Osaka et al., 1980) and infants (Papaiconomou et al., 2004), who possess a large dural venous plexus and whose arachnoid granulations are not fully developed until 4–6 months of age (Mack et al., 2009). This notion is further supported by recent studies showing postnatal development of meningeal lymphatic vessels (Antila et al., 2017). Alternatively, these areas exhibiting signs of dual (blood/lymphatic) vessel identity could reflect fluctuations in vascular/endothelial cell phenotype plasticity controlled by the equilibrium between three endothelial cell fate regulatory genes (Notch, COUP-TFII, and Prox1) as described by Kang et al. (2010). In any instance, whether and what physiological role they play remains to be investigated.

**FIGURE 4:**
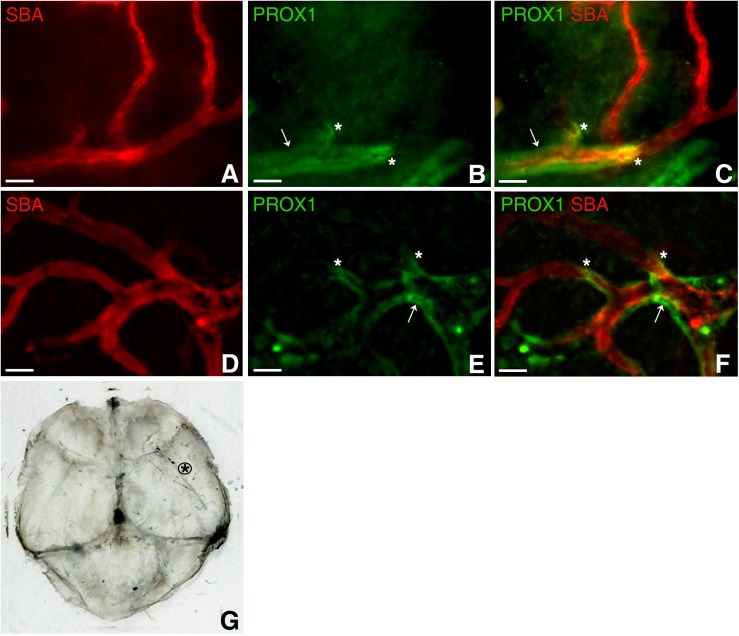
Dural paravascular lymphatic structures. To visualize the blood vasculature, Prox1eGFP mice, in which lymphatic vessels exhibit bright green fluorescence, the hearts were perfused with Kreb’s/albumin solution containing AlexaFluor 594-conjugated SBA lectin. Spinning disk confocal microscopy revealed regions of the blood vasculature (SBA lectin, red) accompanied by dural paravascular PROX1-positive structures (white arrows). After PROX1-positive structures terminate (white asterisks), the blood vessels proceed further through the tissue. In **(A,D)** SBA lectin blood vessel staining (red). In **(B,E)** Prox1eGFP lymphatics staining (green). **(C,F)** Superimposed image. **(G)** Black asterisk approximates the dura mater area where images were taken. Scale bars in A through F 50 μm.

To add to the complexity of the vascular system of this hitherto perceived “non-functional” protective covering of the brain, PROX1-positive structures were identified around arterioles several order off of the middle meningeal artery, which were identified as such based on vessel architecture and their connection with the branches of the known major arterial vessel (Middle Meningeal Artery), in dura mater parietal area (Figure 4) approximated in Figure 4G. Our impression is that these formations appear similar to the paravascular structures described by Iliff et al. (2012) in the brain. However, whether these are indeed similar structures performing the same function, or these are lymphatic vessels flanking the arterioles require further investigation. We found that these formations are restricted to a particular sheet (or sheets) of a multilayered connective tissue structure (Protasoni et al., 2011). They accompany large segments of blood vessel and terminate (Figures 4B,C,E,F, asterisks) while blood microvessels progress further through the tissue free of the surrounding PROX1-positive structures (Figure 4).

**FIGURE 5:**
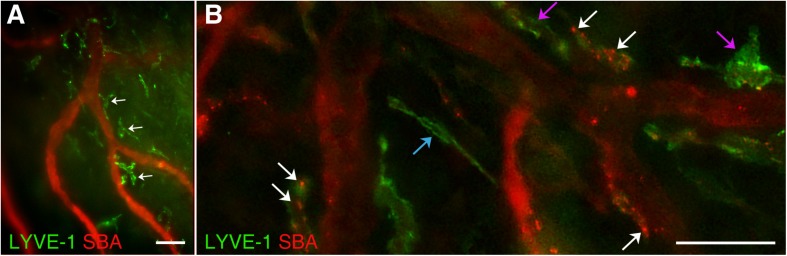
LYVE-1 positive macrophage-like structures. In wild type C57BL/6J mice, blood vasculature and lymphatics were visualized by AlexaFluor 594-conjugated SBA lectin and AlexaFluor 488-conjugated anti-mouse Lyve-1 antibody, respectively. **(A)** Numerous LYVE-1 positive blood vessel-associated macrophage-like structures (green, white arrows) were found throughout the dura connective tissue stroma. **(B)** LYVE-1 positive structures (green) appearing as individual vessels (blue arrow) or macrophage-like structures closely associated with blood vessels (purple arrows) including LYVE-1 positive areas with SBA staining on their walls (white arrows) are observed. In **(A,B)** LYVE-1 staining (green) and SBA lectin staining (red). Scale bars 50 μm.

This study has revealed abundant macrophage-like LYVE-1 positive structures around arterial vessels throughout the dura connective tissue stroma (Figure 5A). Morphologically, they appear similar to those, previously described by Maruyama et al. (2005, 2007) in cornea. These structures are most likely formed by specific subsets of macrophages and connect to the systemic circulation as they actively accumulate dye introduced by intracardiac injection. However, how these stained cells located in DM stroma communicate with the systemic circulation needs to be determined. Previously, it was shown that under inflammatory condition in the corneal stroma, LYVE-1^+^ cells of macrophagic origin are capable of creating tube-like constructs involved in the formation of lymphatic vessels thus contributing directly to lymphangiogenesis (Maruyama et al., 2005, 2007). It was concluded that these macrophages were essential for vascular repair and remodeling (Liu et al., 2016) as well as reducing tissue edema (Ji, 2005). We posit that a subset of dura mater resident macrophages with similar function could play a surveillance role communicating between the blood and lymphatic circulations of the dura. Interestingly, to date we only detected these macrophage-like structures with LYVE-1 staining in wild type animals, but could not observe them in Prox1^eGFP^ mice. Based on this, we speculate that these structures could be LYVE-1 expressing F4/80-positive macrophages, which do not express Prox1 (Schledzewski et al., 2006; Cho et al., 2007; Choi et al., 2011). Morphologically, these LYVE-1 positive structures also appear similar to Mato’s fluorescent granular perithelial (FGP) cells, a specific macrophage lineage cells surrounding brain arterioles, which “also function as a component of blood brain barrier and play a critical role in scavenger functions in the central nervous system” (Mato et al., 1996; Ookawara et al., 1996). Furthermore, yet another population of isolated “mural lymphatic endothelial cells” that surround meningeal blood vessels was recently identified in zebrafish (Bower et al., 2017). These cells that form by sprouting from blood vessels express lymphatic endothelial cell markers and are distinct from endothelial cells, pericytes, macrophages, or astrocytes. They express proangiogenic growth factors and appear to be essential for normal meningeal vascularization (Bower et al., 2017). It is also possible that these are LYVE-1 positive macrophages that closely associate with arterial blood vessels and participate in the control of blood vessel stiffness, as it was most recently described by Lim et al. (2018).

Our study demonstrates that dura mater LYVE-1 positive structures could be seen as individual lymphatic vessels (Figure 5B, blue arrow) or closely associated with blood vessels macrophage-like structures (Figure 5B, purple arrows). Interestingly, some of the pachymeningeal LYVE-1 positive structures (Figure 5B, white arrows) have areas of SBA staining on their walls, which may indicate the ability for vessel plasticity or points of specialized communication between the two systems.

In summary, while confirming recent observations from other laboratories regarding the existence of dura mater lymphatics (Aspelund et al., 2015; Louveau et al., 2015), this study reveals for the first time several additional noteworthy and novel findings extending our understanding of the complexity of a hitherto underappreciated tissue. (1) We demonstrate the presence of diverse and abundant non-sinus-associated lymphatic structures of varied sizes that exist autonomously or in close association with small arterial blood vessels throughout dura mater. (2) Paravascular lymphatic structures found in cranial dura appear to be restricted to particular sheets of connective tissue stroma. The functional significance of these structures requires additional investigation. (3) Microvascular structures with dual systemic/lymphatic endothelial identity suggest specialized areas within cranial dura mater indicative of the unique dura mater microenvironment bringing the systemic blood circulation and lymphatics into close association. We posit that these structures may facilitate fluid exchange between the two compartments representing the means by which cerebral spinal and interstitial fluid is channeled and separated from macromolecules and waste products routed to the cervical lymph nodes. Understanding the anatomical features of blood and lymphatic pachymeningeal vessels, their relationship and plasticity under physiological and pathological conditions is an important goal of modern microvascular and neurophysiology research.

## Ethics Statement

All animal experimental procedures were approved by the University of Missouri Institutional Animal Care and Use Committee.

## Author Contributions

OG, VH, LX, FB, KP, and VG contributed to the conception and design of the work, acquisition, analysis, and interpretation of data, drafting the work and revising the article critically for important intellectual content, and final approval of the version to be published.

## Conflict of Interest

The authors declare that the research was conducted in the absence of any commercial or financial relationships that could be construed as a potential conflict of interest. The handling editor declared a shared affiliation, though no other collaboration, with the authors at the time of review.
