# Supplementary material for: Complex Non-sinus-associated Pachymeningeal Lymphatic Structures: Interrelationship With Blood Microvasculature
Source: Front Physiol. 2019 Oct 31;10:1364. doi: 10.3389/fphys.2019.01364 (PMC6834776; doi:10.3389/fphys.2019.01364)
Supplement: Supplementary file 1 [file Data_Sheet_1.PDF]

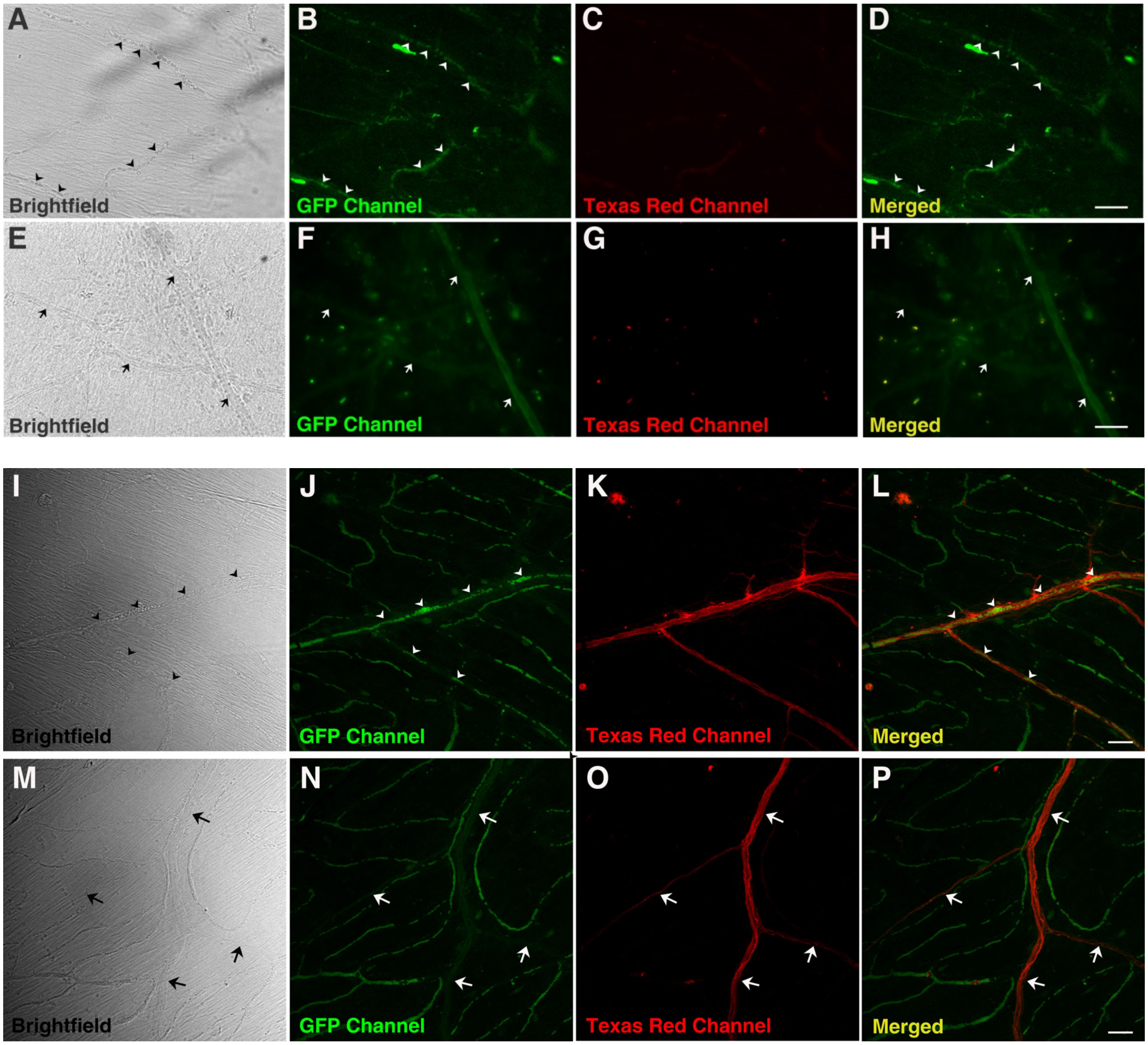

Supplementary Figure 1.

Auto fluorescence controls. A through D, Dura mater of the non-perfused C57BL/6J wild type mouse was completely isolated from the skull, prepped, fixed and permeabilized exactly as experimental samples, however was not stained with any fluorophores. Flat mounts were prepared and mounted on a slide using ibidi Mounting Medium for fluorescence microscopy and imaged using the same filter cubes and settings as for experimental samples. In A, a bright field image shows the presence of blood cells (black arrowheads) remaining in the lumen of systemic blood vessels. In B and D, these same cells (white arrowheads) exhibit bright auto fluorescence similar to the artefacts observed in Figs. 1, D through F in the manuscript main body. This problem is eliminated by transcardial perfusion of the mouse prior to dura mater isolation and processing. In E through H, immediately following euthanasia, wild type mouse was perfused with 2.5 ml of prewarmed to 37°C Kreb's/BSA solution in PBS. Dura mater was completely isolated from the skull and prepped as experimental samples, but was not stained with any

fluorophores. Flat mounts were prepared and mounted on a slide as above and imaged using the same filter cubes and settings. In E, please note the absence of blood cells within the lumens of systemic blood vessels (black arrows) on the bright field image. In F and H, please note only weak green auto fluorescence of the same vascular structures shown in E (white arrows), but no auto fluorescent blood cells are detectable. In C and G, the auto fluorescence in Texas Red channel is practically negligible. In PROX1eGFP model (I through P), in order to distinguish between GFP positive lymphatic vessels and auto fluorescent blood cells present in systemic blood vessels, the latter were counterstained with wheat germ agglutinin (WGA) lectin conjugated with AlexaFluor 594. Multiple blood cells, which could be observed within the lumens of systemic blood vessels on bright field image (black arrowheads in I), exhibit bright green auto fluorescence (white arrowheads in J and L) similar to the artefacts seen in the Fig. 1, B of the manuscript main body. These artefacts are completely absent in blood vessels void of blood cells (black arrows in M, white arrows in N through P) and the blood vessels themselves exhibit only weak green auto fluorescence (white arrows in N). Scale bars in D, H, L and P, 50 μm.
